# Zoonotic Potential of Chronic Wasting Disease after Adaptation in Intermediate Species

**DOI:** 10.3201/eid3012.240536

**Published:** 2024-12

**Authors:** Tomás Barrio, Sylvie L. Benestad, Jean-Yves Douet, Alvina Huor, Séverine Lugan, Naïma Aron, Hervé Cassard, Juan Carlos Espinosa, Alicia Otero, Rosa Bolea, Juan María Torres, Olivier Andréoletti

**Affiliations:** Unité Mixte de Recherche de l’Institut National de Recherche pour l’Agriculture, l’Alimentation, et l’Environnement 1225 Interactions Hôtes-Agents Pathogènes, École Nationale Vétérinaire de Toulouse, Toulouse, France (T. Barrio, J.-Y. Douet, A. Huor, S. Lugan, N. Aron, H. Cassard, O. Andréoletti); Norwegian Veterinary Institute, Ås, Norway (S.L. Benestad); Consejo Superior de Investigaciones Científicas, Madrid, Spain (J.C. Espinosa, J.M. Torres); Universidad de Zaragoza, Zaragoza, Spain (A. Otero, R. Bolea)

**Keywords:** prions, prion disease, chronic wasting disease, European moose, transmission barrier, enhanced zoonotic potential, transgenic model, zoonoses

## Abstract

Chronic wasting disease (CWD) is an emerging disease in Europe. We report an increase in interspecies transmission capacity and zoonotic potential of a moose CWD isolate from Europe after passage in an ovine prion protein–expressing host. Those results indicated some CWD prions could acquire enhanced zoonotic properties following adaptation in an intermediate species.

Chronic wasting disease (CWD) is a highly contagious prion disease affecting members of the Cervidae family. CWD is widely spread across North America, where it endangers the survival of free-ranging cervid populations. In Europe, CWD was reported in a reindeer (*Rangifer tarandus tarandus*) from Norway in 2016 (*1*). Since 2016, several cases have been reported in Norway, Sweden, and Finland in multiple species, including reindeer, red deer (*Cervus elaphus*), and moose (*Alces alces*) (*2*).

Whereas CWD strains circulating in North America exhibit some uniformity (*3*), the cases found in Europe are more variable. Transmission into rodent models has revealed multiple CWD strains that are apparently different than strains in North America, and moose cases in Norway have demonstrated biochemical patterns distinct from previous cases in Europe (*4*). We characterized the interspecies transmission potential of 1 moose CWD isolate from Norway (Norwegian Veterinary Institute identification no. 16–60-P153) (*4*) by intracerebral injection of mouse models expressing the normal prion protein (PrP^C^) sequences from several species (Figure, panel A).

We anesthetized and inoculated 6-to-10-week-old mice with 2 mg of equivalent tissue (20 µL of 10% brain homogenate) in the right parietal lobe. We monitored the inoculated animals daily and humanely euthanized animals at the onset of clinical signs or after the preestablished endpoint of 700 days postinfection (dpi). We conducted a systematic proteinase K­–resistant prion protein (PrP^res^) detection by using Western blot.

Inoculation of the original CWD isolate did not cause the propagation of detectable prions in Tg340 mice expressing methionine (TgMet) or Tg361 mice expressing valine (TgVal) at position 129 of human PrP^C^. We did not observe PrP^res^ in brain tissue or disease occurrence in bovine PrP^C^-expressing mice (BoTg110) after intracerebral inoculation of the CWD isolate (Table; Figure, panel B).

**Table:** Transmission of a moose CWD isolate in a study of zoonotic potential of chronic wasting disease after adaptation in intermediate species*

Model characteristics	TgMet				TgVal					Tg338				BoTg110		
	No./ no_._†	Mean dpi (SD)	PrP^res^ band type		No./ no_._†		Mean dpi (SD)	PrP^res^ band type		No./ no_._†	Mean dpi (SD)	PrP^res^ band type		No./ no_._†	Mean dpi (SD)	PrP^res^ band type
Prion strains																
M1^CJD^ (sCJD MM1)																
1st passage	6/6	219 (17)	21 kDa		6/6	327 (19)‡		21 kDa		ND				ND		
2nd passage	6/6	239 (8)‡	21 kDa		6/6	286 (16)‡		21 kDa		ND				ND		
V2^CJD^ (sCJD VV2)																
1st passage	6/6	618 (81)‡	21 kDa		6/6	168 (12)‡		19 kDa		ND				ND		
2nd passage	6/6	509 (41)‡	21 kDa		6/6	169 (12)‡		19 kDa		ND				ND		
Classical BSE																
1st passage	1/12	739‡	BSE§		0/12		>750‡	NA		6/6	>750¶	BSE§		6/6	295 (12)#	BSE§
2nd passage	9/12	613 (43)‡	BSE§		0/12		>750‡	NA		6/6	682 (52)¶	BSE§		6/6	265 (35)#	BSE§
Sheep-adapted BSE																
1st passage	6/6	690 (83)#	BSE§		ND					6/6	>750¶	BSE§		6/6	254 (19)¶	BSE§
2nd passage	5/5	564 (39)#	BSE§		ND					6/6	653 (32)¶	BSE§		6/6	234 (12)¶	BSE§
Tg338-adapted BSE																
1st passage	6/6	596 (92)	BSE§		0/6	>700		BSE§		5/5	224 (37)	BSE§		6/6	222 (22)	BSE§
2nd passage					ND					ND				ND		
Moose CWD (16–60-P153)																
1st passage	0/12	>700	NA		0/12	>700		NA		2/12	612, 717	19 kDa, 21 kDa		0/12	>700	NA
2nd passage	0/6	>700	NA		ND					5/5	167 (4)**	21 kDa		ND		
	ND				ND					6/6	244 (33)††	21 kDa		ND		
Tg338-adapted moose CWD‡‡																
1st passage	1/8	561	19+21 kDa		5/6	483 (35)		21 kDa		7/7	95 (5)	21 kDa		5/5	431 (32)	20 kDa
2nd passage	ND				4/4	311 (12)		21 kDa		ND				ND		

*Results show moose isolate (16–60-P153) from Norway and reference prion strains (human sCJD strains M1CJD and V2CJD, cattle strain c-BSE) in transgenic mouse models expressing human PrPC 129M (TgMet) and 129V (TgVal), ovine VRQ PrPC (Tg338) and bovine PrPC (BoTg110). BSE, bovine spongiform encephalopathy; dpi, days post-inoculation; NA, not available; ND, not done; PrP, prion protein; PrP^res^, PK-resistant prion protein; VRQ, valine136-arginine154-glutamine171 ovine PrP^C^ variant. †No. affected mice/total no. inoculated. ‡Transmissions reported in (*5*) §Transmissions reported in (*6*) ¶Transmissions reported in (*7*) #Transmissions reported in (*8*) **Transmission was performed from the brain of the first-passage mouse that showed a 19 kDa banding pattern. ††Transmission was performed from the brain of the first-passage mouse that showed a 21 kDa banding pattern. ‡‡The Tg338-adapted isolate corresponds to the brain of a second-passage Tg338 mouse that was culled at 170 dpi after infection with first-passage 19K brain.

**Figure:**
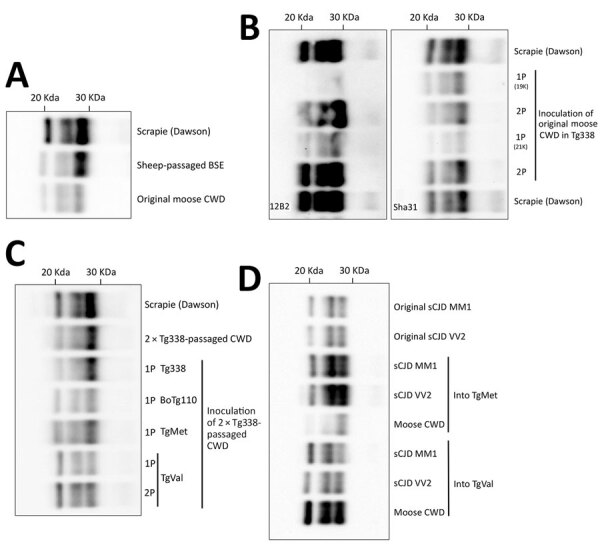
Western blot analysis in a study of zoonotic potential of chronic wasting disease after adaptation in intermediate species. Results show PK-resistant PrP (PrP^res^) banding patterns of a moose CWD isolate from Europe after transmission to transgenic PrP models. A) Original 16–60-P153 CWD isolate compared with reference Dawson and sheep-passaged BSE. B) Transmission of the original moose CWD isolate to mice ovine PrP^C^ genotype VRQ (Tg338), resulting in positive transmission with either 19 or 21 kDa PrP^res^ on first passage and 21 kDa PrP^res^ on second passage; PrP Sha31 antibodies (right panel) and PrP 12B2 antibodies (left panel). C) Transmission of Tg338-adapted (second passage) moose CWD isolate to Tg338, BoTg110, TgMet, or TgVal. D) Comparison of PrP^res^ banding patterns in TgMet and TgVal inoculated with the Tg338-adapted moose CWD isolate or with M1^CJD^ and V2^CJD^ reference sCJD strains. Immunodetection was performed by using either the Sha31 antibody to detect the amino acid sequence YEDRYYRE (145–152), or the 12B2 antibody to detect the amino acid sequence WGQGG (89–93). Dawson (a reference 21-kDa scrapie strain) is included on all panels except D for molecular weight reference. 1P, 1st passage; 2P, 2nd passage; BoTg110, bovine PrP^C^-expressing mice; BSE, bovine spongiform encephalopathy; CJD, Creutzfeldt-Jakob disease; CWD, chronic wasting disease; PK, proteinase K; PrP, prion protein; PrP^C^, normal prion protein; PrP^res^, PK-resistant prion protein. TgMet, Tg340 mice expressing methionine; TgVal, Tg361 mice expressing valine.

We inoculated the CWD isolate in Tg338 mice, which overexpress ovine PrP ≈8 times. At 612 and 717 dpi (Table), 2 of 12 animals showed clinical signs of prion disease, and we detected PrP^res^ accumulation in their brain tissue (Figure, panel B). Of note, the 2 animals showed different PrP^res^ banding patterns, with the nonglycosylated band migrating to 19 kDa in the first mouse and to 21 kDa in the second. Both PrP^res^-containing brains transmitted disease with 100% efficacy to second-passage Tg338 mice, which contained 21-kDa PrP^res^ in their brains (Figure, panel B). A third passage resulted in the incubation period shortening (95 ± 5 dpi). Our observations are consistent with a progressive adaptation of the moose CWD prion to the ovine-PrP^C^ expressing model and suggest moose CWD prions in Europe may have an intrinsic capability to propagate in ovine species with the VRQ genotype.

We next determined whether adaptation of this moose CWD agent to Tg338 altered its capacity to cross species barriers. For that purpose, we inoculated Tg338-adapted moose CWD prions (passaged twice in Tg338) to the same panel of PrP^C^-expressing mice models. Inoculation of the Tg338-adapted isolate to BoTg110 resulted in 100% disease transmission that showed a banding pattern and intermediate molecular weight from 19–21 kDa (Figure, panel C; Appendix Figure) and an incubation period of 431 ± 32 dpi (Table), which suggested the lack of a major transmission barrier. In addition, 1 of 8 inoculated TgMet mice showed clinical signs at 561 dpi (Table). PrP^res^ in the brain of that mouse was revealed by a mixed 19 + 21–kDa banding pattern (Figure, panel C). A second passage in TgMet is underway.

Inoculation of TgVal resulted in efficient transmission (5/6 animals); the mean incubation period was 483 ± 35 dpi (Table) and accumulation was 21-kDa PrP^res^ (Figure, panel C). On second passage, transmission was 100% and we observed a shorter incubation period (311 ± 12 dpi).

The incubation periods and PrP^res^ biochemical profiles of the CWD prions that propagated in the TgMet and TgVal mice greatly differed from those observed in mice inoculated with the most prevalent human prion strains or with classic bovine spongiform encephalopathy (BSE), sheep-adapted BSE, or Tg338-adapted c-BSE (Table; Figure, panel D). Those results might suggest this CWD-derived prion strain differs from other strains documented in those models. Further investigation is necessary.

The evolution of moose CWD zoonotic potential after its passage in an ovine PrP^C^-expressing host is reminiscent of the well-documented altered capacities of the c-BSE agent to cross the human species barrier after adaptation in sheep and goats (*9*). The codon 129-dependent response to infection of humanized mice with Tg338-adapted CWD is also compatible with studies demonstrating the role of this polymorphism in susceptibility to prions (*10*).

In summary, our results demonstrate the potential capacity of some CWD agents to transmit to sheep or other farmed animals. Our results highlight the need to experimentally assess and monitor this transmission risk under natural exposure conditions. In addition, the dramatic changes of the zoonotic capacity of the CWD isolate we documented from Europe clearly demonstrate the risk adaptation and propagation of cervid prions into farmed animals represents. Although additional studies are needed to characterize these emerging agents, our findings have major potential implications for animal and public health.

Appendix Additional information about zoonotic potential of chronic wasting disease after adaptation in intermediate species.
