# Supplementary material for: Zoonotic Potential of Chronic Wasting Disease after Adaptation in Intermediate Species
Source: Emerg Infect Dis. 2024 Dec;30(12):2691–4. doi: 10.3201/eid3012.240536 (PMC11616668; doi:10.3201/eid3012.240536)
Supplement: Appendix — Additional information about zoonotic potential of chronic wasting disease after adaptation in intermediate species. [file 24-0536-Techapp-s1.pdf]

Article DOI: https://doi.org/10.3201/eid3012.240536

EID cannot ensure accessibility for supplementary materials supplied by authors. Readers who have difficulty accessing supplementary content should contact the authors for assistance.

Zoonotic Potential of Chronic Wasting Disease After Adaptation in Intermediate Species

Appendix

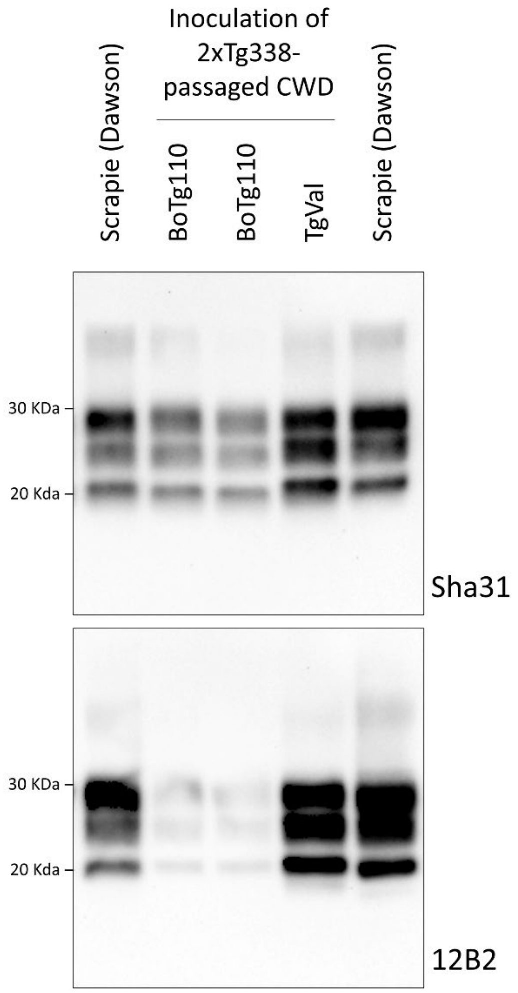

Appendix Figure. Comparison of PK-resistant PrP (PrP^{res}) banding patterns with anti-PrP antibodies Sha31 and 12B2 after transmission of Tg338-adapted (two passages) to BoTg110 and TgVal. Dawson (a reference 21-kDa scrapie strain) is showed for molecular weight reference; the arrow on the left side of each image corresponds to 21 kDa MW.
